# Fatal Effects of Ether Vapour on Animals

**Published:** 1847-04

**Authors:** 


*Fatal Effects of Ether Vapour on Animals.*—M. Gruby found, in his experiments on ether-vapour, that dogs twenty days old lost their sensibility in from eighteen to twenty minutes. Grown-up dogs lost the power of sensation in eight minutes, and died if the action of the ether was continued for forty-five minutes. The dogs recovered their sensibility and motion when they were exposed to the air, if the experiment with the ether was not prolonged beyond eighteen minutes for the young, and forty to forty-four minutes for the adults. Young dogs, which had ceased to breathe, were brought to life by copious bleeding from the jugular vein.—*Ibid.*

